# Agricultural Production Space Suitability in China: Spatial Pattern, Influencing Factors and Optimization Strategies

**DOI:** 10.3390/ijerph192113812

**Published:** 2022-10-24

**Authors:** Yuxin Pan, Yuancheng Lin, Ren Yang

**Affiliations:** School of Geography and Planning, Sun Yat-Sen University, Guangzhou 510275, China

**Keywords:** agricultural production space, resource environmental carrying capacity, agricultural regional planning, agricultural sustainability, China

## Abstract

The paper uses the analytic hierarchy process (AHP), spatial autocorrelation analysis, and geographic detectors to reveal the spatial pattern of agricultural production space suitability in China, explores the impact mechanism of agricultural production development, and explores the optimization and promotion strategies for the development of regional agricultural production in various regions in the future. The results show that the resource and environmental carrying capacity, and the agricultural production space suitability under the direction of China’s agricultural production function, show a ‘polarization’ development trend in space, with high levels in the southeast and low levels in the northwest, with significant spatial agglomeration. The factors influencing the suitability of agricultural production have significant spatial differentiation laws in the Nine Agricultural Areas of China. Climate change factors are the dominant factors affecting the areas with poor resource endowment and traditional agricultural areas in the northwest. Factors that reflect the level of urbanization are the main factors that affect the agricultural production space suitability in the middle and lower reaches of the Yangtze River and South China. China’s agricultural production spatial suitability areas can be divided into nine types of suitable geographical areas. In the future, the impacts of climate change and urbanization on agricultural production space should be considered, and strategies should be taken, according to local conditions, in different regions to improve their suitability.

## 1. Introduction

For a long time, the one-sided pursuit of economic growth in urban development, the extensive development mode of urban construction land expansion and the one-way unbalanced flow of urban and rural factors have greatly squeezed the production space of agriculture [1]. At the same time, frequent global warming, El Niño and other extreme climate changes have caused global temperature and precipitation imbalance [2]. Agricultural production space is faced with multiple practical difficulties, such as space compression, intensified external disturbances, loss of agricultural production entities, and insufficient input of production factors, which seriously restrict the development and transformation of agricultural production [3,4]. The changes in suitability of agricultural production space has become an important practical issue of general planning concern for international organizations, such as the Intergovernmental Panel on Climate Change (IPCC) [5], the Food and Agriculture Organization of the United Nations (FAO) [6] and the United Nations Sustainable Development Goals (SDGs) [7,8]. In this context, as the main spatial carrier of food security and farmers’ livelihoods, the issue of how to accurately identify the suitability of agricultural production space and explore its influencing factors has become key to promoting the development of agricultural and rural modernization and promoting the new pattern of agricultural production [9].

Agricultural production space is a complex system, affected by social, economic, cultural, institutional and other dimensions, as well as the interaction of national and local top-down multi-scales [10,11]. The existing research on the suitability of agricultural production space has carried out in-depth analysis on the indicator system [12], evolution process [13] and driving factors [14] of the suitability of agricultural production space from the national, provincial, municipal, county, town and village scales [15]. The research area involves key ecological function areas [16], black soil cultivation areas [17], karst landform areas [18], industrial towns [19] and economically developed coastal cities [20], and other special natural economic development areas. However, previous studies have focused more on considering the impact of single natural or socio-economic factors, such as natural conditions, and input and output on the suitability of agricultural production space, ignoring the impact of urbanization and climate change on the suitability of agricultural production space. Climate conditions are the basis of, and necessary conditions for, agricultural production, and they also greatly affect the spatial production layout of agriculture [21]. In recent years, the frequent occurrence of the effects of climate change and extreme weather events have posed a great threat to agricultural production, and have had a great negative impact on agriculture and food security in developing countries [22]. Climate change largely affects the temporal and spatial pattern of natural resources, such as land resources and water resources, on which agricultural production depends [23,24]. It can damage agricultural production by destroying the agricultural labor force and hindering the input of production factors [25]. For example, scholars found that the increase of high temperature days restricted the growth and development of crops, leading to a serious reduction in crop yield in inland subtropical regions [26,27]. In addition, rapid urbanization and industrialization have led to the rapid expansion of urban construction land, which has greatly squeezed rural space and agricultural production space, and has also brought a lot of ecological and environmental problems [28,29]. Urban expansion is closely related to the decline of agricultural land use intensity, which has caused great pressure on natural resources, especially in economically developed coastal areas [30]. At the same time, technological progress brought about by urbanization has also improved agricultural output. The improvement of agricultural production efficiency has avoided the blind expansion of agricultural production, and further enhanced the sustainability of agricultural production, on the basis of ensuring the production capacity of agricultural land per unit [31]. On the other hand, the use of a large amount of agricultural machinery has produced a large number of greenhouse gases and anthropogenic aerosols, which has led to the increase of potential evapotranspiration of crops in local arid and semi-arid areas, and the continuous expansion of arid areas of agricultural production [32,33]. In general, the existing research has not paid attention to the overall impact of climate change and urbanization on the suitability of agricultural production space.

China is the world’s largest food producer and also the country with the fastest urbanization rate. In the context of climate change and urbanization, studying the suitability of China’s agricultural production space has important enlightenment significance for agricultural production and layout of other developing countries in the world, and can provide useful experience for coordinating urbanization and agricultural development. This paper attempts to identify the dynamic evolution process of agricultural production space suitability in China by constructing a comprehensive index evaluation system for agricultural production space suitability evaluation, and analyzing the impact mechanisms and optimization strategies of different scales, locations and types of agricultural production space. This paper seeks to fill the gap in the existing research on the suitability of agricultural production space, which has neglected the impact of climate change and urbanization. By filling this gap, this paper helps in explaining and understanding the evolution trend and law of agricultural production space in China, and provides a reference for the optimization and adjustment of agricultural space and sustainable agricultural development in the future.

The main structure of this paper is as follows: Section 2 introduces the research design, including the index system, research methods, data sources and research region; Section 3 show the research results, explain the spatial differentiation and influencing factors and regional types of differentiation and optimization strategies of agricultural production space suitability in China; Section 4 is the discussion; Section 5 summarizes the research conclusions, introduces the research insufficiency, and proposes a forecast to future research.

## 2. Materials and Methods

### 2.1. Research Methods

#### 2.1.1. The Index System of Agricultural Production Space Suitability

In order to analyze the agricultural production space, this paper first constructs the evaluation index of agricultural production space suitability. Based on the conceptual definition and understanding of the spatial suitability of agricultural production [34,35,36], the agricultural production space suitability in China is based on the results of an evaluation of the resource and environmental carrying capacity, superimposing the spatial form, geographic conditions and production conditions, including five indicators (excepting resource and environmental carrying capacity). In this paper, the AHP method was used to establish an indicator system for the agricultural production space suitability with a complete system, a clear hierarchy and a clear structure [37]. The evaluation levels, such as the target level, the criterion level, and the scheme level, respectively, were determined (Table 1), and a pairwise comparison and judgment on the relative importance relationship within the same level and with the indicators at the previous level was conducted, so as to construct a judgment matrix. Testing was performed Through the random consistency test value (Consistency Index, CR).

#### 2.1.2. The Influencing Factors of Agricultural Production Space Suitability

In order to analyze the relationship between the suitability of agricultural production space and urbanization and climate change, this paper starts from the connotation of urbanization and climate change, and combines the relevant research on the impact of urbanization and climate change on the suitability of agricultural production space [38]. Six indicators, such as population density and water and heat imbalance index, were selected, and quantitatively explored in different regions (Table 2). The influencing factors of agricultural production space suitability and the influencing mechanism is deeply explored.

#### 2.1.3. Exploration Model of Influencing Factors on Spatial Suitability of Agricultural Production

In this paper, geodetectors are used to analyze the factors affecting the spatial suitability of agricultural production. Geodetection is a quantitative research method that detects spatial differentiation characteristics and reveals the driving factors and influencing factors behind spatial differentiation [39,40], and is currently widely used in land use, meteorology, public health, regional economy and planning, tourism, geological landforms, ecological environment, remote sensing and computer networks. The mathematical logic behind the use of a geodetector is to assume that a region can be divided into several sub-regions, and to distinguish the spatial differentiation by identifying the relationship between the Total Sum of Squares (SST) of the entire region and the Within Sum of Squares (SSW) of the variances of each sub-unit. If the overall variance of the region is greater than the sum of the variances of the sub-regions, there is heterogeneity in the space, which is measured by the *q*-statistic. The calculation formula is as follows:

(1) $$q=1-\frac{1}{N\sigma^{2}}\sum_{h=1}^{L}N_{h}\sigma_{h}^{2}=1-\frac{SSW}{SST}$$

(2) $$SSW=\sum_{h=1}^{L}N_{h}\sigma_{h}^{2},\;SST=N\sigma^{2}$$

In the formula, *h* = 1, …, *L* is the number of secondary regions. $N_{h}$ and N are the number of space units in the subunit and the whole area, respectively, and $\sigma_{h}^{2}$ and $\sigma^{2}$ are the variances of the subunit and the whole area, respectively.

The threshold range of *q* is [0,1]. When *q* = 0, it indicates that the spatial distribution of agricultural production space suitability in China is in a random distribution state. An increase of *q* shows that the influence factors of independent variables have a greater impact on agricultural production space suitability in China. This paper selected five indicators, population density, night light index, added value of secondary production, water and heat balance index, and annual average temperature, to detect the impact of urbanization and climate change on the spatial pattern of agricultural spatial suitability in China.

#### 2.1.4. Regional Type Division of Agricultural Production Space

According to the regional conditions, development orientation, resource and environment carrying capacity, suitability degree of agricultural production space and other factors of different agricultural production spaces, and referring to the traditional naming method of regional types [41], this paper divided the regional type characteristics of agricultural production spaces with the three-stage naming method of “geographical location + leading influencing factors of agricultural production space suitability + degree of agricultural production space suitability in 2018”. The national regional space was divided into several different regional types, and corresponding optimization strategies were proposed.

### 2.2. Data Sources

The social and economic data, such as the fertilizer application amount and the total power of agricultural mechanization, in the comprehensive evaluation system of agricultural production space suitability were mainly acquired from the *China Statistical Yearbook* (*County-Level*). The mean elevation was derived from the SRTM 90 m DEM Digital Elevation Database. The soil erosion degree and the spatial distribution index data of China’s annual 1 km vegetation index (NDVI) were obtained from the website of the Resources and Environment Science Data Center, Chinese Academy of Sciences (http://www.resdc.cn, accessed on 23 September 2022). The Chinese water system vector shp data came from the National Geomatics Center of China (https://www.ngcc.cn, accessed on 28 September 2022). Meteorological data, such as precipitation, accumulated temperature, and temperature, were derived from the China Regional Surface Meteorological Element-Driven Dataset of the National Tibetan Plateau/Third Pole Environment Data Center (http://data.tpdc.ac.cn, accessed on 2 October 2022). The land use data were derived from the China Multi-period Land Use Land Cover Remote Sensing Monitoring Dataset (CNLUCC). The traffic data came from the geographic data sharing service platform (http://geodata.pku.edu.cn, accessed on 4 October 2022) of the College of Urban and Environment Sciences, Peking University. The vegetation net primary productivity data (Net Primary Productivity, NPP) came from the Google Earth Engine platform (https://lpdaac.usgs.gov, accessed on 7 October 2022).

### 2.3. Nine Agricultural Areas of China

**Figure 1:**
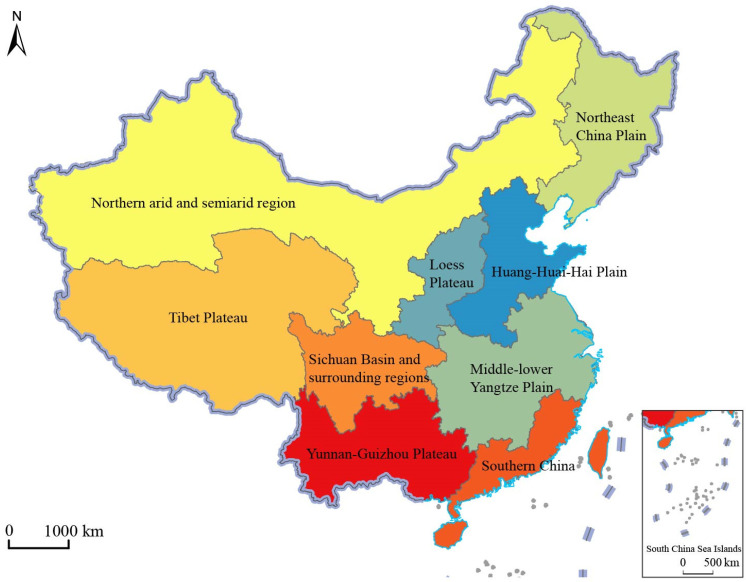
Nine Agricultural Areas of China.

The Nine Agricultural Areas of China reflect agricultural development conditions, agricultural production conditions and agricultural spatial patterns in a certain period, and are the result of a planning and implementation process that integrates elements of nature, society, economy and ecology [42,43]. Its fundamental purpose is to solve the subjective and objective contradiction between human development and agricultural development, and to seek the optimal allocation and layout of agricultural productivity in a certain region [44,45]. For a long time, after the exploration of Chinese geographers, a unified opinion on China’s agricultural division has been basically formed, and a relatively extensive understanding has been formed. According to the background conditions of regional agricultural production and the future development orientation, the whole region of China is divided into Nine Agricultural Areas, including the Northeast China Plain, Northern arid and semi-arid region, Huang-Huai-Hai Plain, Loess Plateau, Qinghai Tibet Plateau, Middle–Lower Yangtze Plain, Sichuan Basin and surrounding regions, Yunnan-Guizhou Plateau, and Southern China (Figure 1).

## 3. Results

### 3.1. Spatial Differentiation Characteristics

**Figure 2:**
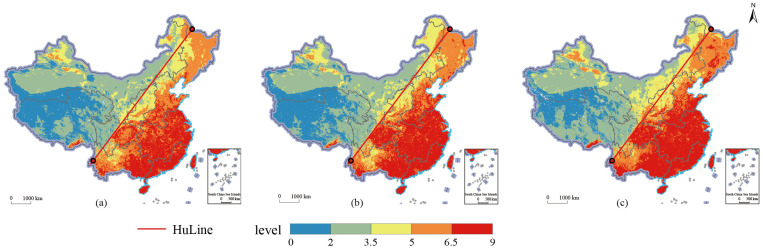
Spatial distribution of agricultural production space suitability in 2000, 2009 and 2018. (**a**) agricultural production space suitability in 2000; (**b**) agricultural production space suitability in 2000; (**c**) agricultural production space suitability in 2018.

The spatial distribution characteristics of the suitability of agricultural production space were obtained by overlaying and processing the grid data of the indicators of the spatial suitability of agricultural production from Table 1 in Arcgis and performing spatial visualization (Table 3, Figure 2). In general, the proportion of each region in each suitability degree in 2000, 2009 and 2018 did not change much and was relatively stable. Specifically, the areas with poor suitability were mainly located in the Qinghai Tibet Plateau and arid and semi-arid areas in the north. These two major agricultural areas accounted for 64.66% and 33.19%, 66.43% and 32.03%, 63.82% and 34.34% of all poor suitability in 2000, 2009 and 2018, respectively. The arid and semi-arid areas in the north were the main components of the regions with poor suitability and showed a decreasing trend, accounting for 70.69%, 70.68% and 60.57%, respectively. However, the proportion of the Qinghai-Tibet Plateau in the poor suitability areas gradually increased, accounting for 18.84%, 20.56% and 31.35%, respectively. The regions with general suitability were still dominated by the northern arid and semi-arid regions, and the proportion was gradually increasing, accounting for 56.36%, 70.68% and 70.02%, respectively. From 2000 to 2009, there was a large increase (14.32%) in, and proportion of, the Northeast Plain. Loess Plateau, Huang-Huai-Hai Plain and Yunnan-Guizhou Plateau gradually decreased from 17.61% in 2000, 13.27%, 5.69% and 4.16%, and had shrunk to 10.65%, 11.67%, 3.60% and 1.51% by 2018. In the regions with higher suitability, except for the northeast plain region, which had a high proportion and strong volatility, there was expansion from 40.06% (2000) to 46.01% (2009) and then shrinking to 38.99% (2018). The proportions were relatively uniform, with little variation. The areas with the best suitability were mainly distributed in the middle and lower reaches of the Yangtze River, the Yunnan-Guizhou Plateau, South China, the Huang-Huai-Hai Plain, the Sichuan Basin and surrounding areas and other plains and coastal areas. Among them, the proportion of the middle and lower reaches of the Yangtze River and South China gradually decreased, with a decrease of 8.46% and 4.54%, respectively, from 2000 to 2018. The proportions of the Huang-Huai-Hai Plain and the Northeast Plain increased slightly, from 9.71% and 1.00% in 2000 to 13.14% and 2.89% in 2009, and then to 13.64% and 6.94% in 2018. The increase in precipitation in the middle and high latitudes improved the suitability of agricultural planting in this region to a certain extent.

### 3.2. Influencing Factors of Spatial Differentiation

**Figure 3:**
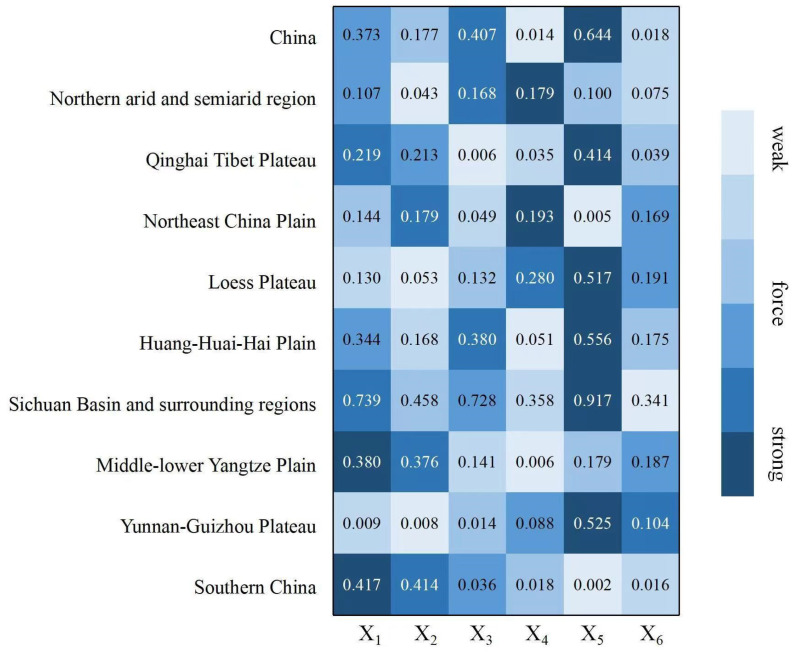
Comparison of the forces of detection factors in nine agricultural areas.

In this paper, six potential influencing factors, including population density (X_1_), night light index (X_2_), land-average secondary industry added value (X_3_), water–heat imbalance index (X_4_), average annual temperature (X_5_), and crop water demand (X_6_), were detected. The matching degree of spatial characteristics was analyzed for four first-level suitability indicators, including resource environmental bearing capacity (Y_1_), spatial form (Y_2_), location conditions (Y_3_), and production conditions (Y_4_). In addition, based on the detection of influencing factors, this paper selected the three most powerful factors for each first-level index of suitability for spatial matching (Table 4). The agricultural production space suitability is affected by the comprehensive disturbance of multi-dimensional factors, such as climate change and urbanization. The *q* statistics of each influencing factor from large to small were the annual average temperature (0.644), the average secondary production value added (0.407), population density (0.373), night light index (0.177), crop water requirements (0.018) and water–heat imbalance index (0.014). The agricultural production space suitability in China was mainly affected by factors such as the average annual temperature, the average industrial added value of the land, and the population density (Figure 3).

**Figure 4:**
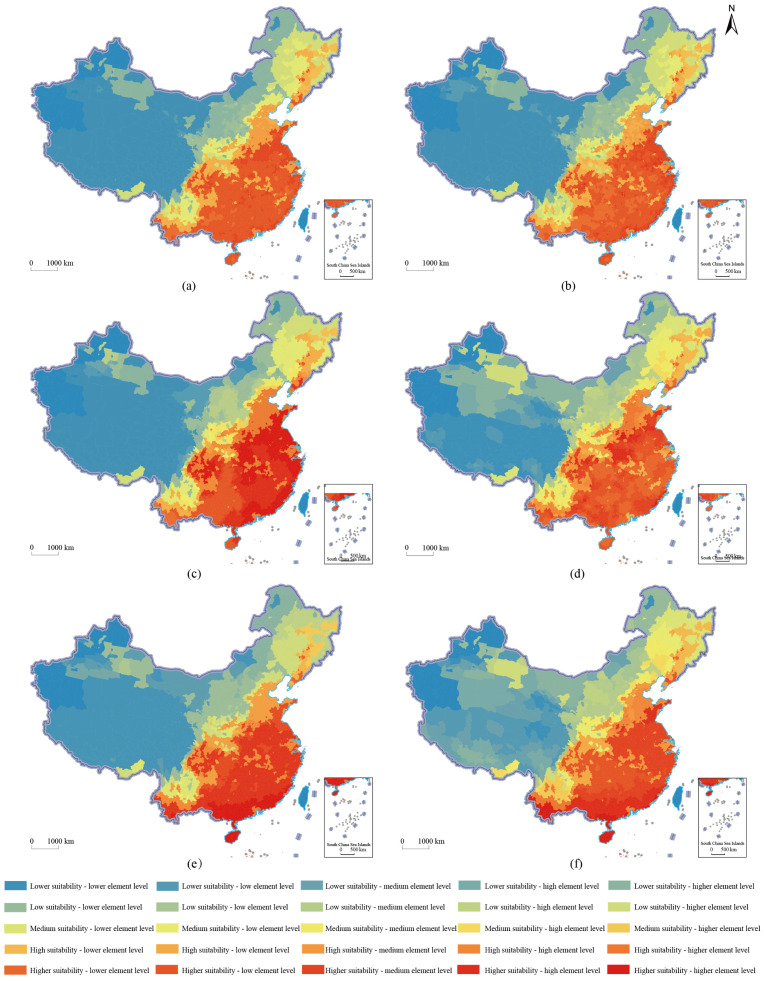
Distribution of agricultural production space suitability and spatial matching of factors in China in 2018. (**a**) population density; (**b**) night light index; (**c**) land-average secondary industry added value; (**d**) water-heat imbalance index; (**e**) average annual temperature; (**f**) crop water demand.

The agricultural production space suitability is subject to the comprehensive disturbance of multi-dimensional factors, such as climate change and urbanization. This paper selected three indicators, population density, night light index and land-average added value of secondary production, which reflect the development level of urbanization, and reflect the degree of climate change. The water–heat imbalance index, the average annual temperature and the crop water demand, and a total of 6 indicators, were used to conduct in-depth exploration and research on the formation mechanism of the spatial pattern of agricultural production space suitability in China. Using the classification of the agricultural production space suitability in China and the classification results of each influencing factor, coupling matching analysis was carried out, and the distribution results of the agricultural production space suitability in China, and the spatial matching of elements in 2018, were obtained (Figure 4). The *q* statistics of the factors affecting the agricultural production space suitability in China, from large to small, were annual average temperature (0.644), land-average secondary production added value (0.407), population density (0.373), night light index (0.177), crops’ water demand (0.018) and hydrothermal imbalance index (0.014). The agricultural production space suitability was mainly affected by factors such as the average annual temperature, the average industrial added value of the land, and the population density.

**Figure 5:**
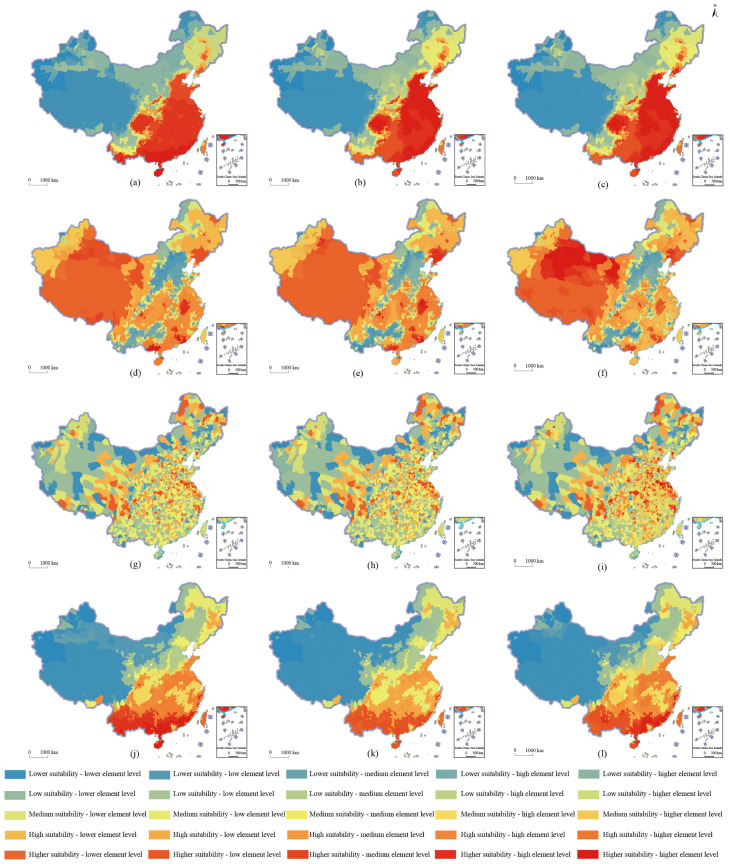
Spatial matching distribution of each first-level index and factor of agricultural production space suitability in China in 2018. (**a**) average annual temperature; (**b**) land-average secondary industry added value; (**c**) population density; (**d**) average annual temperature; (**e**) land-average secondary industry added value; (**f**) water-heat imbalance index; (**g**) population density; (**h**) night light index; (**i**) land-average secondary industry added value; (**j**) average annual temperature; (**k**) population density; (**l**) land-average secondary industry added value.

In order to deeply explore the formation and influence mechanism of suitability, this paper also detected the force *q* statistic for each influencing factor and the first-level indicators of resource and environmental carrying capacity, spatial form, location conditions and production capacity. The spatial matching degree between the top 3 influencing factors of the force and the 4 first-level indicators was analyzed (Figure 5). Climatic factors, such as annual average temperature, were the fundamental influencing factors affecting regional resources and environment carrying capacity (0.622), cultivated land form (0.201), and vegetation ecological capacity (0.421). Factors such as the added value of the secondary industry per land, population density and other factors were also important factors that affected the suitability of the location, production and resource background conditions of agricultural production space.

### 3.3. Regional Types of Agricultural Production Space in China

**Figure 6:**
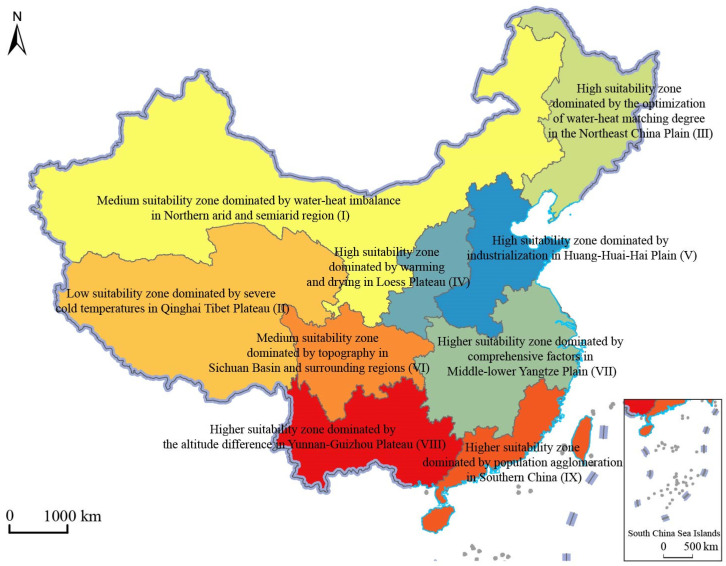
The division of regional types of agricultural production space in China.

Based on the influencing factors above, the whole country was divided into the following zones: Medium suitability zone, dominated by water–heat imbalance in Northern arid and semi-arid region (I); Lower suitability zone, dominated by severe cold temperatures in Qinghai Tibet Plateau (II); Higher suitability zone, dominated by the optimization of water–heat matching degree in the Northeast China Plain (III); Higher suitability zone, dominated by warming and drying in Loess Plateau (IV); Higher suitability zone, dominated by industrialization in Huang-Huai-Hai Plain (V); Medium suitability zone, dominated by topography in Sichuan Basin and surrounding regions (VI); High suitability zone, dominated by comprehensive factors in the Middle–lower Yangtze Plain (VII); High suitability zone, dominated by the altitude difference in Yunnan-Guizhou Plateau (VIII); High suitability zone, dominated by population agglomeration in Southern China (IX) (Figure 6). An optimization promotion strategy is proposed according to its suitability-driven characteristics.

### 3.4. Regional Pattern Characteristics and Optimization Strategies

The differences in resource endowment, socio-economic conditions, location conditions, and government policies, of the different types of agricultural areas resulted in different suitability characteristics and development paths among the various types of agricultural areas. There were also significant differences in the mathematical characteristics of resource and environmental carrying capacity conditions, spatial form conditions, location conditions, production conditions, and suitability characteristics (Table 5). Based on the comprehensive analysis, and the characteristics of the macro background values of the nine agricultural areas, optimization and promotion strategies are given for the future agricultural production space in different regions according to local conditions (Table 6).

## 4. Discussions

### 4.1. Natural Environmental Conditions Are the Basic Factors Affecting the Suitability of Agricultural Production Space in China

Land resources, water resources, climate conditions and environmental conditions are the basic factors that affect agricultural production and play a decisive role in the suitability of agricultural production space. First of all, climate change leads to changes in planting systems and crop production layout. Global warming has led to an increase in the temperature in middle and high latitudes of China, leading to an earlier crop sowing date, a later winter season, an overall extension of the growing season, and an increase in the accumulated temperature during the winter, which may lead to changes in planting systems. More northern and high-altitude regions can carry out multi-cropping production and planting, and the planting areas expand northwards. The multiple cropping index increased, and the suitable planting area was further expanded [46]. Secondly, climate change leads to the change of grain production capacity. Climate warming and the increase of precipitation leads to the movement of a large number of crop planting areas to high latitudes and altitudes. The vegetation coverage in northern China and alpine terrain shows an increasing trend. Winter wheat and other major grain crops show a trend of moving north and expanding west, which could increase the unit yield of grain crops. Thirdly, climate change may bring meteorological disasters and disturbances of pests and diseases. Climate change is often accompanied by extreme disasters, such as mud rock flows, landslides and drought, which bring incalculable losses to local agricultural production. In addition, the rise of temperature increases the accumulated temperature during the winter, which is conducive to the overwintering of pests and diseases, providing a hotbed for the occurrence of pests and diseases, and adversely affecting agricultural production and planting. Finally, climate change brings disturbance to other industries, such as animal husbandry. Rising temperature, increasing accumulated temperature, and decreasing snowfall and snow cover make grassland areas warm and dry. On the one hand, this phenomenon is conducive to livestock overwintering, but a warm and dry climate is not conducive to the growth of pasture [47].

### 4.2. Economic and Social Development Are Peripheral Factors Affecting the Suitability of Agricultural Production Space in China

The rapid progress of urbanization and industrialization has brought China many conveniences, such as economic development, social progress and industrial upgrading, as well as positive and negative effects on the suitability of agricultural production space. First of all, the urbanization process has brought a lot of agricultural transfer population, and the modern non-agricultural sector has absorbed a large amount of labor from rural areas, which has brought about the non-agricultural transfer of agricultural production subjects and optimized the allocation of urban and rural resource elements. Secondly, the rapid progress of urbanization has led to extensive and disorderly expansion of construction land, which has had a far-reaching impact on agricultural production subjects and space, leading to reduction of the quantity and quality of cultivated land, the occupation of high-quality cultivated land and permanent basic farmland, and great reduction in the suitability of agricultural production space. For example, in the middle and lower reaches of the Yangtze River, where there is a high degree of economic and social development, and, likewise, in the southeast coastal areas, agricultural production space has become fragmented and decentralized due to the interference of high intensity human activities; thus, affecting the suitability of agricultural production space. Finally, urbanization has driven the development of urban industry and technological progress, and has had multiple effects on agricultural production space. On the one hand, the development of urban industry drives the innovation of agricultural technology. Industrialization provides more advanced and convenient mechanized production tools for agricultural production, effectively saves the labor input cost of agricultural production [48], improves the efficiency of agricultural production, and further promotes the large-scale centralized business model. The factor flow has brought a lot of investment, advanced technology and development opportunities to rural areas in the process of urban–rural transfer, but, on the other hand, it has also led to a lot of industrial transfer pollution, polluted river systems and human settlements in rural areas, which have not been conducive to the organization of agricultural production, and have reduced the suitability of agricultural production space.

### 4.3. Market and Policy Factors Are Social Factors Affecting the Suitability of Agricultural Production Space in China

In addition, price mechanism, livelihood mode, policy guarantee and technology input are also auxiliary factors that affect the suitability of agricultural production space in China. First, the market price mechanism affects farmers’ willingness to plant, and the fluctuation of market price affects the income of agricultural producers engaged in agricultural production activities, thus, affecting the enthusiasm of agricultural producers engaged in agricultural production activities. This then affects the way agricultural production entities engage in agricultural production and operation activities, causing changes in the quality of agricultural production space, and, ultimately, leading to changes in the suitability of agricultural production space. Second, the shift of the main agricultural production body’s part-time livelihood mode is also an important factor affecting the suitability of agricultural production space. Areas such as the middle and lower reaches of the Yangtze River and South China have conditions conducive to production, such as high matching of water and heat resources, flat terrain for farming, and abundant labor force. However, these areas often have a high level of economic development, and the labor income of central cities is significantly higher than that of rural areas. Farmers, based on the consideration of rational economic people, tend to move to cities and the surrounding areas to engage in other occupations with higher returns. As a result, a large number of agricultural production subjects, mainly young and middle-aged people, have turned to non-agricultural production modes, which has caused problems, such as aging and feminization of agricultural production subjects [49], and, thus, reducing the suitability of agricultural production space. Third, good agricultural policies enhance the suitability of agricultural production space. The central and local governments have improved the enthusiasm of agricultural production entities by introducing policies and regulations, such as the project of returning farmland to forests and grasslands, ditch management and land reclamation, and the guarantee mechanism of compensation and incentives, which have promoted the adjustment of the agricultural production structure, the restoration of soil fertility and the restoration of the ecological environment, and optimized and improved the suitability of agricultural production space. Fourthly, the application of modern technology has a two-way impact. The rational application of modern technology can effectively improve the suitability of agricultural production space. The input of agricultural technology and resources has become the main driving force for agricultural expansion and production [50]. However, excessive, or unreasonable, application of modern technologies, such as pesticides and fertilizers, have a negative impact on agricultural production space.

## 5. Conclusions

Based on the background of the era of urbanization, industrialization and climate change. and the major strategic needs of rural revitalization, sustainable agricultural development and food security, this paper integrated multidisciplinary theories and research methods, and deeply developed the spatial and temporal pattern, evolution process, influencing factors, type of differentiation and coping strategies of agricultural production space suitability in China. The specific conclusions are as follows:

First, the proportion of each region in each suitability degree did not change much and was relatively stable in 2000, 2009 and 2018. The areas with poor suitability are mainly located in the Qinghai-Tibet Plateau and the northern arid and semi-arid regions, which have improved a lot in the past 10 years, mainly due to the increase in precipitation in the middle and high latitudes, which has improved the suitability of agricultural planting in this region. From the perspective of the spatial differentiation pattern, the agricultural production space suitability in 2000, 2009 and 2018 showed a polarized distribution, with high levels in the southeast and low levels in the northwest. The agricultural production space suitability in each county in China is significantly affected by the suitability of neighboring counties, showing a significant positive correlation.

Second, the factors influencing the agricultural production space suitability in China have significant spatial differentiation laws in the nine agricultural areas in China. Climate change factors, such as annual average temperature and the water–heat imbalance index, are the dominant factors affecting areas with poor resource endowment and traditional agricultural areas in the northwest. Factors that reflect the level of urbanization, such as population density and nighttime light index, are the main factors that affect the agricultural production space suitability in the middle and lower reaches of the Yangtze River and South China with relatively high levels of economic development, good water and heat matching, and favorable location conditions.

Third, based on geographical location, the dominant influencing factors of the agricultural production space suitability, the degree of agricultural production space suitability in 2018, and the types of agricultural production space suitability in China can be divided into the following zones: Medium suitability zone, dominated by water–heat imbalance in Northern arid and semi-arid region; Lower suitability zone, dominated by severe cold temperatures in Qinghai Tibet Plateau; Higher suitability zone, dominated by the optimization of water–heat matching degree in the Northeast China Plain; Higher suitability zone, dominated by warming and drying in Loess Plateau; Higher suitability zone, dominated by industrialization in Huang-Huai-Hai Plain; Medium suitability zone, dominated by topography in Sichuan Basin and surrounding regions; High suitability zone, dominated by comprehensive factors in Middle-lower Yangtze Plain; High suitability zone, dominated by the altitude difference in Yunnan-Guizhou Plateau; High suitability zone, dominated by population agglomeration in Southern China.

Fourth, agricultural production is a production process that is highly dependent on climatic resources, and, at the same time, is subject to comprehensive disturbances by its production subject and other related subjects. Differences in resource endowment, socio-economic conditions, location conditions, and government policies have resulted in different suitability characteristics and development paths among various types of agricultural areas. The natural conditions, environmental conditions and location conditions of regional agriculture reflect the macro background value of agricultural development in the region. The scientific application of strategies, such as the rational development and utilization of regional agricultural resources, the improvement of acquired technologies and policy guidance, are “late remedy” measures and important approaches for the rational utilization of regional agricultural resource conditions and sustainable agricultural development. In the future, optimization and improvement strategies should be given for the agricultural production space in different regions according to local conditions.

This paper enriches the empirical research on spatial adaptability of agricultural production and agricultural sustainable development. It also provides important reference for agricultural regional planning and high-quality agricultural development in other developing countries. Limited by the availability of data, the frequency of natural disasters, farmers’ livelihood choices and market food prices have not been considered, which is also the shortcoming of the paper. As climate change and human activities disturb the agricultural production space more and more intensely, more and more experts and scholars have begun to consider how agricultural production can reduce the frequency of external disturbances and improve the system resilience of agricultural production to actively respond to disturbances. On the one hand, the indiscriminate use of agricultural chemicals worldwide in the last century has increased crop yields to a certain extent, but, at the same time, has brought great damage to terrestrial and aquatic ecosystems. Nanomaterials, biomass charcoal and nitrogen fertilizer, and other new materials, could reduce the disturbance of agricultural production to the outside world. They could also improve the diversification of crops, organic agricultural production, large-scale planting and irrigation in water-deficient arid and semi-arid areas. The resilience of agricultural production could be improved by means of technological upgrading and other means. Therefore, it is urgent to classify the agricultural production space caused by different influencing factors, and to explore the future agricultural development path of each regional type.

## Figures and Tables

**Table 1:** Indicator weights and classification thresholds of agricultural production space suitability.

Criteria	Alternatives	Evaluation Threshold	Score	Quantitative Method
Resource environmental bearing capacity (0.439)	Bearing capacity level (0.439)	Excellent (V)	9	Select indicators such as slope elevation, soil erosion degree, river network density, and non-point source pollution degree to comprehensively evaluate the resource and environmental carrying capacity under the direction of agricultural production functions from the conditions of land resources, water resources and environmental conditions.
		Good (IV)	7	
		Average (III)	5	
		Fair (II)	3	
		Poor (I)	0	
Spatial Form (0.104)	Cultivated land concentration (0.104)	Excellent (>60%)	9	According to the degree of centralized distribution of cultivated land within a certain range, the lower the degree of concentration, the worse the suitability.
		Good (50~60%)	7	
		Average (40~50%)	5	
		Fair (30~40%)	3	
		Poor (<30%)	1	
Geographic condition (0.146)	Cultivated convenient level (0.110)	Excellent (0~6 min)	9	Use GIS road accessibility analysis, calculate the range that can be reached within 30 min from the county road when driving at a speed of 20 km/h. The longer the time, the lower the accessibility and the worse the suitability.
		Good (6~12 min)	7	
		Average (12~18 min)	5	
		Fair (18~24 min)	3	
		Poor (24~30 min)	1	
	Degree of proximity to rivers (0.036)	Excellent (<5 km)	9	Use GIS spatial analysis tools to measure the distance from the center line of the river. The farther the distance, the lower the proximity and the worse the suitability. In addition, the radiation range of rivers of different levels should be considered.
		Good (5~10 km)	7	
		Average (10~30 km)	5	
		Fair (30~50 km)	3	
		Poor (>50 km)	1	
Production condition (0.311)	Vegetation production capacity (0.233)	Excellent (>1 kgC/m^2^/year)	9	GIS spatial analysis tools were used to calculate the net contribution of organic matter to photosynthesis per unit area of green plants in each year. The lower the NPP value, the less the accumulation of organic matter and the worse the regional suitability.
		Good (0.7~1 kgC/m^2^/year)	7	
		Average (0.4~0.7 kgC/m^2^/year)	5	
		Fair (0.2~0.4 kgC/m^2^/year)	3	
		Poor (<0.2 kgC/m^2^/year)	1	
	Agricultural mechanization level (0.078)	Excellent (>500 MW)	9	Using the collaborative Kriging spatial interpolation tool in GIS, the density of cultivated land is used as collaborative data to correct the data on the degree of agricultural mechanization. The lower the degree of mechanization, the weaker the suitability of the land.
		Good (200~500 MW)	7	
		Average (100~200 MW)	5	
		Fair (50~100 MW)	3	
		Poor (<50 MW)	1	

Note: The contents in brackets in the first two columns are the corresponding index weights. When constructing the weight judgment matrix, use the random consistency test value (Consistency Index, CR) to test, and the CR value is 0.0454, which has passed the consistency test. The score comes from the 1–9 scale method, and the weight comes from the eigenvector calculated by the judgment matrix. As the resource and environment carrying capacity is the bottom-line constraint condition for suitability evaluation, the area with poor resource and environment carrying capacity is scored as 0.

**Table 2:** Detecting indexes of influencing factors of agricultural production space suitability.

Primary Indicator	Secondary Indicators	Specific Contents
Urbanization	Human activity intensity	Population density
	Urban expansion intensity	Night light index
	Degree of industrial development	Secondary production added value/administrative area
Climate change	Degree of water and heat matching	Hydrothermal Imbalance Index
	Temperature change	Average annual temperature
	Sensitivity of crops to moisture	Crop water requirement

**Table 3:** Mathematical characteristics of agricultural production space suitability in nine agricultural areas.

Region Type	Suitability Index in 2000		Suitability Index in 2009		Suitability Index in 2018	
	Level	Mean	Level	Mean	Level	Mean
Northern arid and semi-arid region	Lower	3.135	Lower	3.233	Medium	3.516
Qinghai Tibet Plateau	Low	1.996	Lower	2.025	Lower	2.189
Northeast China Plain	Higher	5.292	Higher	5.609	Higher	5.672
Loess Plateau	Medium	4.406	Medium	4.901	Higher	5.127
Huang-Huai-Hai Plain	Higher	5.759	Higher	6.127	Higher	6.319
Sichuan Basin and surrounding regions	Medium	4.410	Medium	4.691	Medium	4.857
Middle-lower Yangtze Plain	High	6.746	High	6.993	High	7.063
Yunnan-Guizhou Plateau	Higher	6.135	Lower	2.025	High	6.683
Southern China	High	7.138	High	7.305	High	7.368

**Table 4:** Influencing factors and influencing factor detection of suitability level 1 indicators.

*q*-Statistic	X_1_	X_2_	X_3_	X_4_	X_5_	X_6_
Y_1_	0.430 ***	0.252 ***	0.505 ***	0.018 ***	0.622 ***	0.038 ***
Y_2_	0.088 ***	0.071 ***	0.132 ***	0.123 ***	0.201 ***	0.048 ***
Y_3_	0.037 ***	0.034 ***	0.033 ***	0.012 ***	0.028 ***	0.027 ***
Y_4_	0.206 ***	0.058 ***	0.144 ***	0.080 ***	0.421 ***	0.077 ***
Total	0.373 ***	0.177 ***	0.407 ***	0.014 ***	0.644 ***	0.018 ***

Note: *** indicate that the coefficients are significant at the statistical level of 0.01.

**Table 5:** Mathematical characteristics of spatial suitability of agricultural production in nine agricultural area in 2018.

Area Type	Suitability Index	Resource Environmental Bear Capacity	Space Form	Location Conditions	Production Conditions
Medium suitability zone dominated by water–heat imbalance in Northern arid and semi-arid region (I)	3.516 (Medium)	2.131 (Medium)	5.735 (Higher)	5.022 (Medium)	4.262 (Medium)
Lower suitability zone dominated by severe cold temperatures in Qinghai Tibet Plateau (II)	2.189 (Lower)	1.079 (Lower)	7.253 (High)	5.010 (Medium)	2.668 (Lower)
Higher suitability zone dominated by the optimization of water–heat matching degree in the Northeast China Plain (Ⅲ)	5.672 (Higher)	3.675 (Higher)	5.262 (Higher)	5.088 (Medium)	5.845 (Higher)
Higher suitability zone dominated by warming and drying in Loess Plateau (IV)	5.127 (Higher)	3.358 (Medium)	3.640 (Medium)	5.262 (Medium)	5.233 (Medium)
Higher suitability zone dominated by industrialization in Huang-Huai-Hai Plain (Ⅴ)	6.319 (Higher)	4.569 (High)	3.642 (Medium)	5.386 (Medium)	5.839 (Higher)
Medium suitability zone dominated by topography in Sichuan Basin and surrounding regions (Ⅵ)	4.857 (Medium)	2.953 (Medium)	5.568 (Higher)	5.519 (Higher)	5.285 (Medium)
High suitability zone dominated by comprehensive factors in Middle–Lower Yangtze Plain (VII)	7.063 (High)	4.959 (High)	5.010 (Higher)	5.495 (Medium)	0.749 (Low)
High suitability zone dominated by the altitude difference in Yunnan-Guizhou Plateau (VIII)	6.683 (High)	4.209 (High)	4.382 (Medium)	5.059 (Medium)	7.844 (High)
High suitability zone dominated by population agglomeration in Southern China (IX)	7.368 (High)	4.991 (High)	5.094 (Higher)	5.046 (Medium)	7.572 (High)

**Table 6:** Characteristics of regional patterns and optimization strategies of China’s agricultural production space.

Area Type	Resource Environmental Bear Capacity Characteristics	Space Form Characteristics	Location Condition Characteristics	Production Condition Characteristics	Optimization Strategies
Medium suitability zone dominated by water–heat imbalance in Northern arid and semi-arid region (I)	(1) Water–heat resource mismatch (2) Good heat resources and little precipitation	(1) Broad and flat terrain, high concentration of cultivated land (2) Rivers are scarce and tend to become warm and dry (3) Low crop productivity	(1) Low economic development and investment (2) Deep inland and poor transportation (3) Agricultural production space is far away from the residential areas	(1) Poor soil and low soil fertility (2) Agricultural production is relatively concentrated (3) Low agricultural input	(1) Establish a rainwater harvesting project for intensive use of agricultural irrigation water to reshape the spatial and temporal distribution pattern of precipitation resources (2) Establish different models of water harvesting and water irrigation to solve the problem of water use difficulties for producers, livestock, crops, etc., in the agricultural production space (3) The slope could be transformed into a horizontal terrace form
Lower suitability zone dominated by severe cold temperatures in Qinghai Tibet Plateau (II)	(1) High altitude, year-round snow, severely cold climate and insufficient heat (2) Not suitable for the growth of various crops	(1) High terrain and low temperature (2) Steep terrain, low accessibility of agricultural production, inconvenient cultivation	(1) Year-round snow, less temperature changes, less snowmelt, inconvenient use of water resources (2) Water scarcity	(1) Low population density (2) Poor economic development (3) Low mechanization	(1) Following the basic principles of ecological environment protection, convert the restored grasslands and woodlands in the project of returning farmland to forests and grasslands into cultivated land to relieve the pressure on other food-producing areas (2) Appropriately improve agricultural production efficiency in areas where cultivated land resources are concentrated and distributed
Higher suitability zone dominated by the optimization of water–heat matching degree in the Northeast China Plain (Ⅲ)	(1) Global warming increases heat resources (2) Accumulation of crops during wintering and the ability to resist severe cold increase	(1) Flat terrain, fertile land, abundant products, well resource endowment (2) High concentration of cultivated land, suitable for large-scale planting (3) Abundant grain yield	(1) Abundant mineral resources and petroleum resources (2) High agricultural mechanization (3) Mature investment in agricultural modernization technology	(1) Many young and middle-aged laborers (2) The land tends to degrade	(1) Use plastic films and modern ecological materials, and select crop varieties with high cold resistance and long growth period (2) Continue to promote projects, such as returning farmland to forests and grasslands, and high-standard farmland construction (3) Strengthen the supervision of industrial pollution and reduce the excessive use of chemical fertilizers and pesticides
Higher suitability zone dominated by warming and drying in Loess Plateau (IV)	(1) Scarce precipitation, higher evaporation (2) Serious water deficit, climate is warming and drying	(1) Severe erosion (2) Low vegetation coverage and small NPP	(1) Severe soil erosion and general soil degradation (2) Fragile ecological environment	(1) Soft soil, and fine loess particles (2) The soil is fertile and conducive to farming	(1) Update crop varieties, planting structure and production and operation mode (2) Increase investment in agricultural technology and capital investment in ditch control and land reclamation, solve the problem of agricultural irrigation, and improve the efficiency of agricultural production
Higher suitability zone dominated by industrialization in Huang-Huai-Hai Plain (Ⅴ)	(1) High water and heat matching degree, abundant heat resources, light and temperature resources, high production potential (2) Crops are capable of two crops a year	(1) Flat terrain and the vast land (2) It is the second largest alluvial fan plain area in China	(1) It is an important support area for the Beijing-Tianjin-Hebei urban agglomeration (2) High industrial development and the proportion of heavy industry (3) Complete infrastructure construction	(1) High population density, abundant labor force and long history of cultivation (2) An important grain and oil production base in China (3) High agricultural livelihood income	(1) Increase the investment in agricultural mechanization and develop a large-scale and centralized operation mode of agriculture (2) Strengthen the construction of agricultural infrastructure and urban-rural interconnection
Medium suitability zone dominated by topography in Sichuan Basin and surrounding regions (Ⅵ)	(1) The climate is warm and humid, the winter temperature is higher than other areas (2) Less frost, better growing conditions of crops	(1) Rivers are widely distributed (2) Fertile soil	(1) The flood control project is perfect (2) The agricultural water intake is more convenient for irrigation	(1) Good farming culture tradition and development (2) Developed mechanization, mature cultivation technology, cultivated farmers (3) The agricultural development has a path dependence	(1) Gradually return farmland to forest and grassland in steep slope areas to control soil erosion and vegetation destruction (2) Gentle slope areas could be transformed into horizontal terraces (3) Through various means improve unit yield in areas with low production efficiency
High suitability zone dominated by comprehensive factors in Middle-lower Yangtze Plain (VII)	(1) Sufficient water and heat resources (2) Good agricultural production conditions	(1) Flat internal terrain and small undulations (2) The river network is densely covered	(1) Industrialization and urbanization developed early and progressed rapidly (2) Many models of local development have emerged	(1) Well-equipped infrastructure (2) High population density (3) Non-agricultural production space and idleness of land is prominent	(1) Give full play to the geographical advantages, develop diversified and high value-added agricultural production, and build the entire agricultural value chain (2) Develop a new type of agricultural production model with characteristics, and actively introduce multiple subjects to participate (3) Strengthen the planning, remediation and intensification of agricultural production space and land use
High suitability zone dominated by the altitude difference in Yunnan-Guizhou Plateau (VIII)	(1) Abundant heat resources (2) The agricultural development conditions are superior in the south and inferior in the north	(1) Complex terrain and diverse with large fluctuations (2) Poor land resource conditions (3) Land desertification and rocky desertification	(1) Traffic congestion (2) Agricultural production space has less contact with the outside world	(1) High proportion of primary industry in the industrial structure (2) Poor population mobility (3) Sufficient labor	(1) Strengthen the construction of infrastructure, especially the construction of water conservancy facilities (2) Strengthen the policy support of the government (3) Develop tourism agriculture, leisure agriculture, etc.
High suitability zone dominated by population agglomeration in Southern China (IX)	(1) Warm and humid climate (2) Superior water and heat resources (3) Affected by industrialized pollutants, the ecological environment has suffered damage	(1) Flat terrain (2) Densely distributed river network (3) High land fragmented and low intensification, not conducive to large-scale mechanized planting	(1) Adjacent to Hong Kong, Macao and open ports, with a high degree of openness; (2) Industry is mostly export-oriented economy, attracting a lot of investment	(1) High population density (2) High mechanization (3) High investment in agricultural production	(1) Strengthen the transformation of old land and integrate the layout of agricultural space (2) Improve the level of agricultural modernization (3) Construct the entire agricultural value chain of input, production, processing, circulation and retail

## Data Availability

Not applicable.

## References

[1] Alexander J., Ehlers Smith D.A., Ehlers Smith Y.C., Downs C.T. (2021). Urban land development for biodiversity: Suggested development and management guidelines for eco-estates using case studies from coastal Kwazulu-Natal, South Africa. Urban For. Urban Green..

[2] Yang Y.M., Park J.H., An S.I., Wang B., Luo X. (2021). Mean sea surface temperature changes influence enso-related precipitation changes in the mid-latitudes. Nat. Commun..

[3] Spyra M., Kleemann J., Calò N.C., Schürmann A., Fürst C. (2021). Protection of peri-urban open spaces at the level of regional policy-making: Examples from six European regions. Land Use Policy.

[4] Altieri M.A., Nicholls C.I. (2013). The adaptation and mitigation potential of traditional agriculture in a changing climate. Clim. Change.

[5] Qin D.H. (2014). Climate change science and sustainable development. Prog. Geogr..

[6] Food and Agriculture Organization of the United Nations (2016). The State of Food and Agriculture.

[7] Pinstrup-Andersen P. (2009). Food security: Definition and measurement. Food Secur..

[8] Fujimori S., Hasegawa T., Krey V., Riahi K., Bertram C., Bodirsky B.L., Bosetti V., Callen J., Després J., Doelman J. (2019). A multi-model assessment of food security implications of climate change mitigation. Nat. Sustain..

[9] Wang J., He T., Lin Y. (2018). Changes in ecological, agricultural, and urban land space in 1984–2012 in China: Land policies and regional social-economical drivers. Habitat Int..

[10] Campi M., Dueñas M., Fagiolo G. (2020). How do countries specialize in agricultural production? A complex network analysis of the global agricultural product space. Environ. Res. Lett..

[11] Geng S., Shi P., Zong N., Zhu W. (2019). Agricultural Land Suitability of Production Space in the Taihang Mountains, China. Chin. Geogr. Sci..

[12] Zhou J., Fu S., Yang Y., Mao D. (2010). Spatial optimization of agricultural regions under the background of virtual water strategy. Environ. Resour. Yangtze Basin.

[13] Nan G.W., Sun H., Song Y. (2017). Optimization of agricultural production spatial distribution in loess plateau based on virtual water strategy—A case study of Yulin city. Econ. Geogr..

[14] Lu W.C., Mei Y., Li Y. (2008). Regional change in China’s grain production: Effects of labor-land ratio, off-farm employment opportunities and labor compensation. Chin. J. Popul. Sci..

[15] Fu X.Z. (2020). Improving agricultural production suitability evaluation of national land use and space at city and county levels. Planners.

[16] Wang X.L., Wang X. (2018). On the spatial zoning of “urban-agriculture-ecology” In key ecological functional areas based on MOLA method: A case study of Shennongjia forest area. Sci. Technol. Manag. Land Resour..

[17] Gao F.J., Shan P., Ma Q., Han W., Zhou J., Ju T., Wu X. (2017). Spatial autocorrelation of soil moisture and agricultural zoning in a mollisol tillage area of northeast China. J. Nat. Resour..

[18] Lai G.H., Hu B., Li M., Lin S. (2020). Evaluation on spatial suitability of ecological-living-industrial in southwestern guangxi-beibu gulf region. Bull. Soil Water Conserv..

[19] Yu Z.S., Cheng Y., Li X., Sun D. (2020). Spatial evolution process, motivation and reconstruction of “Production-living-ecology” In industrial town: A case study on Qugou town in Henan province. Sci. Geogr. Sin..

[20] Ma L.P., Yang M., Chen J. (2021). Evaluation of the suitability of agricultural space based on multi-source data and integrated learning: Take Zhongshan city as an example. Geomat. Spat. Inf. Technol..

[21] Huyer S., Partey S. (2019). Weathering the storm or storming the norms? Moving gender equality forward in climate-resilient agriculture: Introduction to the special issue on gender equality in climate-smart agriculture: Approaches and opportunities. Clim. Change.

[22] Food and Agriculture Organization of the United Nations (2009). Climate Change and Bioenergy Challenges for Food and Agriculture.

[23] Adger W.N., Barnett J., Brown K., Marshall N., O’Brien K. (2012). Cultural dimensions of climate change impacts and adaptation. Nat. Clim. Change.

[24] Collins M.L. (2013). Climate Change 2013: The Physical Science Basis.

[25] Aryal J.P., Sapkota T.B., Khurana R., Khatri-Chhetri A., Rahut D.B., Jat M.L. (2020). Climate change and agriculture in South Asia: Adaptation options in smallholder production systems. Environ. Dev. Sustain..

[26] Serdeczny O., Adams S., Baarsch F., Coumou D., Robinson A., Hare W., Schaeffer M., Perrette M., Reinhardt J. (2017). Climate change impacts in Sub-Saharan Africa: From physical changes to their social repercussions. Reg. Environ. Change.

[27] Zhao C., Liu B., Piao S., Wang X., Lobell D.B., Huang Y., Huang M.T., Yao Y.T., Bassu S., Ciais P. (2017). Temperature increase reduces global yields of major crops in four independent estimates. Proc. Natl. Acad. Sci. USA.

[28] Nordhaus W. (2019). Climate Change: The Ultimate Challenge for Economics. Am. Econ. Rev..

[29] Bradshaw W.E., Holzapfel C.M. (2006). Evolutionary Response to Rapid Climate Change. Science.

[30] Jiang L., Deng X., Seto K. (2013). The impact of urban expansion on agricultural land use intensity in China. Land Use Policy.

[31] Jaleta M., Baudron F., Krivokapic-Skoko B., Erenstein O. (2019). Agricultural mechanization and reduced tillage: Antagonism or synergy?. Int. J Agric. Sustain..

[32] Chiang F., Mazdiyasni O., AghaKouchak A. (2021). Evidence of anthropogenic impacts on global drought frequency, duration, and intensity. Nat. Commun..

[33] Zhang X., Zwiers F.W., Hegerl G.C., Lambert F.H., Gillett N.P., Solomon S., Stott P.A., Nozawa T. (2007). Detection of human influence on twentieth-century precipitation trends. Nature.

[34] Yu S.-H., Deng W., Xu Y.-X., Zhang X., Xiang H.-L. (2020). Evaluation of the production-living-ecology space function suitability of Pingshan County in the Taihang mountainous area, China. J. Mt. Sci..

[35] Bradley B.A., Estes L.D., Hole D.G., Holness S., Oppenheimer M., Turner W.R., Beukes H., Schulze R.E., Tadross M.A., Wilcove D.S. (2012). Predicting how adaptation to climate change could affect ecological conservation: Secondary impacts of shifting agricultural suitability. Divers. Distrib..

[36] Pérez-Urrestarazu L., Lobillo-Eguíbar J., Fernández-Cañero R., Fernández-Cabanás V.M. (2019). Suitability and optimization of FAO’s small-scale aquaponics systems for joint production of lettuce (*Lactuca sativa*) and fish (*Carassius auratus*). Aquac. Eng..

[37] Lin C.-C., Wang W.-C., Yu W.-D. (2008). Improving AHP for construction with an adaptive AHP approach. Autom. Constr..

[38] Zhou K., Li J., Wang K. (2021). Evaluation on agricultural production space and layout optimization based on resources and environmental carrying capacity: A case study of Fujian province. Sci. Geogr. Sin..

[39] Wang J.F., Xu C.D. (2017). Geodetector: Principle and prospective. Acta Geogr. Sin..

[40] Xu C., Li Y., Wang J., Xiao G. (2017). Spatial-temporal detection of risk factors for bacillary dysentery in Beijing, Tianjin and Hebei, China. BMC Public Health.

[41] Yang R., Pan Y. (2021). Rural vulnerability in China: Evaluation theory and spatial patterns. J. Geogr. Sci..

[42] Deaton B.J., Vyn R.J. (2010). The Effect of Strict Agricultural Zoning on Agricultural Land Values: The Case of Ontario’s Greenbelt. Am. J. Agric. Econ..

[43] Vyn R.J. (2012). Examining for Evidence of the Leapfrog Effect in the Context of Strict Agricultural Zoning. Land Econ..

[44] Xu X., Hou L., Lin H., Liu W. (2006). Zoning of sustainable agricultural development in China. Agric. Syst..

[45] Xu E. (2018). Zoning of Agricultural Resource and Environment in China. Chin. J. Eng. Sci..

[46] Jorgenson D.W. (1961). The development of a dual Economy. Econ. J..

[47] Lv X.M., Zhou G. (2021). A review on ecological and Agro-Meteorology in China. Adv. Meteorol. Sci. Technol..

[48] Zhang Q., Deng Z., Zhao Y., Qiao J. (2008). The impacts of global climatic change on the agriculture in northwest China. Acta Ecol. Sin..

[49] Yi C.J. (2020). Research on the impact of urbanization and industrialization on the growth of agricultural total factor productivity: Evidence based on panel data of 37 counties in Chongqing. J. Chongqing Univ. Soc. Sci..

[50] Tao R., Xu Z., Xu J. (2004). Grain for green project, grain policy and sustainable development. Soc. Sci. China.

